# Optimization, characterization and biosafety of carotenoids produced from whey using *Micrococcus luteus*

**DOI:** 10.1186/s12896-024-00899-6

**Published:** 2024-10-07

**Authors:** Aml A. Hegazy, Samah H. Abu-Hussien, Neima K. Elsenosy, Salwa M. El-Sayed, Mohamed Y. Abo El-Naga

**Affiliations:** 1 Food Science Department, Faculty of Agriculture, Ain Shams University, Cairo, 11241 Egypt; 2 Agricultural Microbiology Department, Faculty of Agriculture, Ain Shams University, Cairo, 11241 Egypt; 3 Genetics Department, Faculty of Agriculture, Ain Shams University, Cairo, 11241 Egypt; 4 Biochemistry Department, Faculty of Agriculture, Ain Shams University, Cairo, 11241 Egypt

**Keywords:** *M. luteus*, Whey, Carotenoid optimization, Antimicrobial activity, Antioxidant properties, Bioprocess safety

## Abstract

This study aimed to optimize the production of carotenoid pigments from *Micrococcus luteus* (ATCC 9341) through the statistical screening of media components and the characterization of antimicrobial, antioxidant, cytogenetic and cytotoxic activities. A BOX-Behnken design was used to assess the effects of whey concentration, inoculum size, pH, temperature, and agitation speed on carotenoid yield. The optimum combination increased production to 2.19 g/L, with a productivity of 0.045 g L^-1^ h^−1^ and a productivity yield of 0.644 g/g, as confirmed by an observed carotene production of 2.19 g/L. The final response surface model fitting the data had an R^2^ of 0.9461. High-performance liquid chromatography (HPLC) analysis identified 12 carotenoid pigment compounds produced by *M. luteus*. The extracts displayed moderate antimicrobial efficacy against Gram-positive bacteria such as *Bacillus cereus* (ATCC 11778), *Staphylococcus aureus* (ATCC 6538), and *E. faecalis* (ATCC 19433), with inhibition zone diameters (IZD) of 29.0, 14.0, and 37.0 mm, respectively, at 1000 μg/mL. However, its effectiveness against Gram-negative bacteria is limited. In comparison, tetracycline exhibited greater antimicrobial potency. The IC_50_ value of carotenoids was used to indicate the antioxidant activity. IC_50_ value from the DPPH assay was 152.80 mg/100mL. An IC_50_ cytotoxicity value greater than 300 μg/mL was found against normal mouse liver cells, with over 68% cell viability even at 300 μg/mL, indicating low toxicity. Histological structure studies revealed normal myocardial muscle tissue, lung tissue, and kidney tissue sections, whereas liver tissue sections revealed ballooning degeneration of hepatocytes and disorganization of hepatic cords. Cytogenetic parameters revealed that the carotene treatment group had a mitotic index (70%) lower than that of the control but higher than that of the positive control, mitomycin, and did not substantially increase numerical (1.2%) or structural aberrations compared with those of the control, suggesting a lack of genotoxic effects under the experimental conditions. In conclusion, optimized culture conditions enhanced carotenoid yields from *M. luteus*, and the extracts displayed promising bioactivity as moderate antibiotics against certain gram-positive bacteria and as antioxidants. The high IC_50_ values demonstrate biosafety. Overall, this bioprocess for enhanced carotenoid production coupled with bioactivity profiling and low cytotoxicity support the application of *M. luteus* carotenoids.

**Supplementary Information:**

The online version contains supplementary material available at 10.1186/s12896-024-00899-6.

## Introduction

In recent years, microbial pigments have attracted increasing scientific attention due to their diverse applications across industries and their promising pharmacological properties [1]. These natural colorants are finding use in sectors such as food, textiles, and cosmetics, while also demonstrating potential in medical applications owing to their antimicrobial, antioxidant, and anticancer properties [2]. Pigment-producing bacteria are ubiquitous in nature, inhabiting diverse ecosystems from freshwater to soil and marine environments. Among the various pigments they produce, carotenoids have garnered special interest for their potential health benefits, including protection against oxidative stress and possible cancer-preventive properties [3]. Notable pigment-producing bacterial genera include *Streptomyces* sp., *Arthrobacter* sp., and *Micrococcus* sp., each capable of synthesizing a range of colorful compounds with unique properties [4]

Carotenoids constitute a diverse group of natural lipid-soluble pigments biosynthesized by plants, algae, fungi, and bacteria that play crucial roles in photosynthesis and nutrition and increase adaptability to ecological niches [5]. Over 750 distinct chemical structures of carotenoids have been identified, contributing to their vast functional diversity. These C40 terpenoid compounds are composed of conjugated double bonds forming an extended polyene system conferring vibrant yellow, orange, and red hues along with singlet oxygen quenching, membrane stabilization, and potent antioxidant activities [6]

Industrially, carotenoids are highly valued for their utility as natural food colorants, feed additives, nutraceuticals, and cosmeceutical ingredients owing to their colouration properties and health-beneficial effects [7]. The global demand for these high-price biomolecules is rapidly increasing, driven by consumer preferences for eco-friendly additives to replace synthetic dyes and the growing commercial market for therapeutics, supplements, and cosmetics to promote wellness [8]. Microbial biotechnology offers a dependable alternative over plant sources for sustainable and renewable carotenoid pigment supplies to cater to these escalating needs through process intensification strategies [9].

Among carotenogenic microbes, *Micrococcus luteus* has attracted attention as a prolific producer of yellow pigments, with zeaxanthin and sarcinaxanthin as major constituents, mediated by the mevalonate pathway [10]. The optimization of cultivation parameters has been shown to increase yields of this Gram-positive bacterium, increasing the prospects for industrial-scale bioprocessing [11]. The utilization of food byproducts such as whey as growth substrates can promote economic benefits through waste recycling while supporting carotenogenesis [12]. Whey proteins provide abundant peptides and amino acids to sustain biomass accumulation and secondary metabolite biosynthesis upon catabolism. However, a high organic load from untreated whey can inhibit microbial growth, warranting systematic pretreatment and component optimization guided by the design of experiments to achieve balanced, productive formulations for carotenoid-enriched biomass generation. It has been noted that whey contains a high concentration of sugars, making it a valuable resource for the industrial creation of various biotechnological products. While numerous modern methods for utilizing whey have been explored, a sustainable approach could involve converting whey through bacterial processes into an affordable fermentation medium for producing natural pigments. Despite often being regarded as waste, whey is nutritionally rich, with lactose, proteins, vitamins, and minerals that can support cell growth. The lactose in whey, being a cost-effective and effective carbon source, is beneficial for different microorganisms [12].

To maximize pigment yield, Response Surface Methodology (RSM) has emerged as a powerful tool in optimizing microbial pigment production, offering a systematic approach to understanding complex interactions between multiple variables. By enabling researchers to efficiently explore and model the relationships between various factors and pigment yield, RSM significantly reduces the time and resources required for optimization experiments, ultimately accelerating the development of cost-effective and scalable production processes [13].

Additionally, microbial carotenoids exhibit a range of biological activities, including antioxidant, antimicrobial, immunomodulatory, anti-inflammatory, anticancer, and chemopreventive properties, which are attributable to their molecular structures, which can modulate redox signalling and membrane dynamics [7]. Elucidating the spectra of the bioactivities displayed is hence vital for directing product development pipelines and specifying targeted applications for these multifunctional biomolecules as pharmaceutical leads, nutraceutical supplements, or active food ingredients [14]. However, biosafety evaluations are critically lacking, with minimal documented cytotoxicity data, hindering regulatory clearances and clinical acceptance [15].

Therefore, this study aims to systematically enhancing carotenoid productivity in whey-grown *M. luteus* using statistical media optimization designed by response surface models, facilitated by Box–Behnken experimental design for screening, and validating the interactive effects of key ingredients and culture parameters. HPLC-based identification of constituent pigments was revealed the diversity of carotenoid species contributing unique bioactive properties. The antioxidant capacity was assessed using a cell-free DPPH radical quenching assay, and the antimicrobial potential was tested against food-borne pathogens. Finally, the cytotoxicity to normal mouse liver cells was evaluated to determine preliminary toxicity risks to evaluate the possibility of recommending their use as food additives.

## Materials and methods

### Microorganism

The *Micrococcus luteus* (ATCC 9341) strain utilized in this research was obtained from the Microbial Resources Center (MIRCEN) in Cairo, Egypt. This bacterial strain was preserved in frozen stocks within nutrient glucose media slants at 4°C and subcultured at monthly intervals. The composition of the nutrient glucose medium was as follows (g/L distilled water): 3.0 g/L beef extract, 5.0 g/L peptone, 10.0 g/L glucose, and 20.0 g/L agar, with the final pH adjusted to 7.2 ± 0.2 at 25°C. To prepare the standard inoculum, 50 mL of nutrient glucose medium in 250 mL Erlenmeyer flasks was inoculated with a full loop of the *M. luteus* culture. The flasks were incubated at 30°C and 120 rpm for 72 h in a rotary shaking incubator (DAIHAN Scientific). This incubation produced a standard inoculum of 7.0 × 10^5^ viable cells/mL, which was used for subsequent shake flask experiments [16]. All pathogenic strains, including *B. cereus* ATCC 11778, *Staph. aureus* ATCC 6538, *E. faecalis* ATCC 19433, *S. typhi* DSM 17058, *S. sonnei* DSM 5570, and *E. coli* ATCC 8739 were also obtained from the Microbial Resources Center (MIRCEN) in Cairo, Egypt.

### Whey powder

#### Commercial spray-dried

Whey powder obtained from Maybi, Turkey (https://www.maybi.com.tr/) with an average composition of 11.2% protein, 0.9% fat, 4% moisture, 70% lactose, and 10.9% ash was used as the growth medium for *Micrococcus luteus* to produce carotenoid pigments.

### Effect of incubation time on carotenoid production by *M. luteus* on whey media

To assess the impact of incubation time on carotenoid pigment production by *M. luteus*, 50 mL of whey broth medium was inoculated with a full loop of the bacterial culture in 250 mL Erlenmeyer flasks. The flasks were then incubated at 30°C and 120 rpm in a rotary shaking incubator (DAIHAN Scientific) for 72 h. Samples of 10 mL were taken from the flasks every 24 h throughout the 72-h period. These samples were centrifuged at 6000 rpm for 15 min at 4°C via a HERMLE (Z 232 k) centrifuge to collect pellets for biomass determination and carotenoid production as described below. Each experiment was conducted in triplicate. This approach enables the evaluation of the effects of incubation time on the growth of *M. luteus* and its carotenoid production via regular sampling and analysis of the culture supernatant over a 72-h incubation period [17].

### Screening of factors affecting (carotenoid pigment) production via Box–Behnken design (BOX)

A Box–Behnken experimental design [18] was employed to explore the combined effects of whey concentration and various process parameters on carotenoid pigment production by *M. luteus*. Five variables were studied at two levels (high and low): whey powder concentration (g/L), pH, inoculum size (%), agitation speed (rpm) and temperature (°C).

The experimental design included 46 unique runs, with the variables set at different combinations of high and low levels as specified by the Box–Behnken matrix. Each run involved inoculating the media with 5–10% standard *M. luteus* inoculum (5.0 × 10^7^ CFU/mL). The inoculated flasks were incubated at 30°C in a shaker incubator, with agitation speeds ranging from 100 to 250 rpm depending on the specific run. After 72 h, both growth and carotenoid production were measured. A second-order polynomial equation was applied to the results to assess the relationships between the variables (whey powder concentration, inoculum size, pH, temperature, and agitation speed) and the response (total carotenoid production). This equation facilitated modelling of the synergistic effects of the variables on carotenoid output. Three-dimensional response surface plots generated from the fitted polynomial equation were used to visualize the relationship between carotenoid production and the experimental levels of each parameter. The Box–Behnken design and analysis helped identify the optimal levels of key process parameters to maximize carotenoid yields from *M. luteus* cultured on whey media.

The design was utilized to examine the synergistic effects of whey as the sole carbon and nitrogen source, along with physical factors such as inoculum size, agitation speed, incubation time, and pH, on carotenoid pigment production. Five variables were selected for this analysis at two levels (high and low), as detailed in Table S1. The chosen variables were the whey concentration, inoculum size, pH, temperature, and agitation speed on the basis of the box matrix design. Table S2 presents the experimental design with the variables and their actual levels. All media were inoculated with 5–10% standard *M. luteus* inoculum (5.0 × 10^7^ CFU/mL). The inoculated flasks were incubated in a shaker incubator (DAIHAN Scientific) at 30°C with shaking speeds ranging from 100 to 250 rpm according to the run number. Growth measurements and total carotenoid pigment production assays were conducted after 72 h of incubation.

Design Expert 12 software was employed to analyse the experimental Box–Behnken design [19]. The response variable, total carotenoid pigment production (Y), was recorded after each experimental run. A second-order polynomial equation was developed through multiple regression analysis to describe the relationships between the response (total carotenoids) and the independent variables (whey powder concentration, inoculum size, pH, temperature, and agitation speed). The polynomial equation was as follows:

$$\text{Y }=\upbeta 0 +\upbeta 1\text{A }+\upbeta 2\text{B }+\upbeta 3\text{C }+\upbeta 11\text{A}2 +\upbeta 22\text{B}2 +\upbeta 33\text{C}2 +\upbeta 12\text{AB }+\upbeta 13\text{AC }+\upbeta 23\text{BC}$$

where Y represents the predicted carotenoid production, β0 is the intercept, β1–3 are linear coefficients, β11–33 are squared coefficients, and β12–23 are interaction coefficients of the independent variables. The goodness of fit of the polynomial model to the experimental data was assessed via the coefficient of determination (R^2^). Three-dimensional response surface plots of the polynomial equation were generated to visualize the interaction between carotenoid production and the experimental levels of each independent variable. In essence, regression modelling and response surface methodology facilitated the optimization and prediction of carotenoid yields on the basis of whey and other process parameters.

### Verification of growth parameters for optimizing carotenoid production by *M. luteus*

To validate the statistical model for optimizing growth and carotenoid production, an experiment was conducted under the optimal conditions predicted by the model [20]. The validation medium was prepared by adjusting the whey medium to pH 7. The medium was inoculated with 7.5% standard inoculum of *M. luteus* (5 × 10^7^ CFU/mL). The flasks were maintained at 32.5°C with shaking at 175 rpm for 72 h. After incubation, growth and total carotenoid pigment levels were analysed via previously described methods. The experimentally obtained values for growth and carotenoid production under the optimized conditions were compared with the expected values predicted by the statistical model. This validation experiment served to confirm the accuracy of the model in predicting the optimum whey powder concentration, inoculum size, pH, temperature, and agitation speed for maximizing *M. luteus* growth and carotenoid yields. Matching the experimental and predicted values indicates that the model can successfully optimize the fermentation process.

### Extraction and determination of total carotenoids

To extract the total carotenoid pigments after fermentation, the cell pellets were macerated in methanol until they became colorless, and the extraction process was conducted in the dark. After that, centrifugation was done at 6000 rpm for 15 min at 4°C to separate the cell biomass from the carotenoid-rich supernatant. The supernatant was collected for total carotenoid quantification, while the cell pellet was used to determine biomass levels. Following extraction, the carotenoid product was sterilized by filtration and then lyophilized to obtain a dried powder for further analysis and studies [13].

### Identification of carotenoid pigments

The initial identification of pigments from symbiotic bacteria was conducted via a visible spectrophotometer (SPECTROstarNano, BMG LABTECH) at wavelengths ranging from 300 to 800 nm. The spectral pattern within the carotenoid range was observed at 300–600 nm [17, 21]. β-carotene served as the reference standard for calculating the total carotenoid content.

### Parameters for total carotenoid pigment production

The carotenoid production parameters were determined via the following equations:

$$\mathrm{Productivity}\;(\mathrm P)\:=\:\mathrm{concentration}\;\mathrm{of}\;\mathrm{carotenoid}\;\mathrm{pigment}\;(\mathrm g/\mathrm L)/\mathrm{fermentation}\;\mathrm{time}\;(\mathrm h)\:=\:\mathrm{mg}/\mathrm L/\mathrm h.$$

Total carotenoid pigment yield coefficient relative to biomass (Yp/x) (g/g) = amount of carotenoid pigment produced (g)/amount of biomass (g/L) [22].

### Carotenoid separation by high-performance liquid chromatography (HPLC)

For HPLC identification, 0.2 g of carotenoid powder was dissolved in 1 mL of HPLC-grade methanol and then centrifuged. The supernatant was used for HPLC analysis via an Agilent 1260 series. Separation was performed with an RP-C18 column (2.5 × 30 cm). The mobile phase consisted of water (A) in acetone (B) (HPLC grade 99.9%) at a flow rate of 0.7 mL/min. Detection was monitored at 280 nm with a multiwavelength detector. Each sample mixture was injected in a 20 µL volume, with the column maintained at 25 °C. UV absorption spectra for both standards and samples were recorded between 200–700 nm. All the solutions and the mobile phase were degassed and filtered through a 0.45 µm membrane filter (Millipore). The retention times and UV absorption spectra of the compounds were compared with those of the standards.

### Antioxidative activity

The DPPH (1,1-diphenyl-2-picrylhydrazyl) radical scavenging ability of microbial carotenoids was assessed as described by [23]. For the DPPH method, 100 μL of a freshly prepared DPPH solution (0.2 μM in methanol) was mixed with 100 μL of carotenoids (1.5–50 mg/100 mL) and left in the dark for 30 min at 24.6 ºC. The mixture's absorbance was then recorded at 517 nm. The scavenging ability of the DPPH (1,1-diphenyl-2-picrylhydrazyl) radical (% inhibition) was calculated via the following equation:

$$(\%\;\mathrm{inhibition})=\frac{Abs\;control-Abs\;sample}{Abs\;control}\times\;100$$

where:

Abs control: is the absorbance of the control without sample.

Abs sample: is the absorbance in the presence of the sample.

### Antimicrobial activity of the produced carotenoids

The minimum inhibitory concentration (MIC) of Micrococcus *luteus* carotenoid extract against various pathogenic bacteria was determined using the agar diffusion method. Two-fold serial dilutions of the carotenoid extract (1000, 500, 250, 125, and 75 µg/ml) were prepared in DMSO and incorporated into nutrient agar (NA) plates. The plates were inoculated with standardized suspensions of test organisms, including Gram-positive (*Bacillus cereus*, *Staphylococcus aureus*, *Enterococcus faecalis*) and Gram-negative (*Salmonella typhi*, *Escherichia coli*, *Shigella sonnei*) bacteria. Tetracycline (1000 µg/ml) served as a positive control, while plates without carotenoid extract were used as negative controls. The inoculated plates were incubated at 30°C for 24 h. After incubation, the inhibition zone diameters (IZD) were measured in millimeters. The MIC was defined as the lowest concentration of carotenoid extract that completely inhibited visible bacterial growth (IZD > 0 mm). Each assay was performed in triplicate, and the results were expressed as mean ± standard deviation [24].

### Toxicological potential of the produced carotenoid pigments

Human skin fibroblast (HSF) cells were obtained from Nawah Scientific, Inc., located in Mokatam, Cairo, Egypt. DMEM supplemented with antibiotics (100 mg/mL streptomycin, 100 U/mL penicillin) and 10% heat-inactivated fetal bovine serum was prepared in a 5% CO_2_ humidified atmosphere at 37°C. Cell viability was evaluated via the sulforhodamine B (SRB) assay method [25].

### Evaluation of chromosomal aberrations in bone marrow cells from albino mice

Twenty male albino mice (25 g ± 2) were obtained from Faculty of Veterinary Medicine, Ain Shams University, Cairo, Egypt. All animals acclimated for one week before receiving oral administration of carotene oil (100 µg/mL) for 15 consecutive days. Two hours prior to sacrifice using cervical disclosure, the mice were given an intraperitoneal injection of colchicine (0.4 mg/kg). Bone marrow from the femur was collected with 0.9% NaCl and centrifuged at 1000 rpm for 10 min, and the pellet was subsequently resuspended in a hypotonic KCl solution (0.56 g/100 mL distilled water) at 40°C for 30 min. The cells were fixed in methanol:glacial acetic acid (3:1 v/v), and the centrifugation and fixation process was repeated at least three times. The cell suspension was spread onto chilled slides, flame-dried, and stained with 10% phosphate-buffered Giemsa (pH 6.8) for 30 min. A minimum of 50 well-spread metaphases per animal were analysed microscopically at 100 X magnification for structural aberrations and numerical changes [26]. Cervical disclosure was performed in accordance with the guidelines established by the Canadian Council on Animal Care (CCAC). The animal was positioned ventral side down on a flat, firm surface. Firm pressure was applied at the base of the skull using the thumb and forefinger, while simultaneously grasping the tail with the other hand. To execute the dislocation, a quick, controlled motion was employed: the thumb and forefinger pushed forward and downward on the skull, while the restraining hand pulled the tail upward at an approximate 30° angle to stabilize the mouse's body. The effectiveness of the procedure was assessed by measuring the time to respiratory arrest in seconds. Euthanasia was deemed successful when the mouse ceased breathing immediately following dislocation (time to respiratory arrest = 0 s).

### Statistical analysis

The data from the aforementioned experiments were analysed via one-way ANOVA with "Design Expert" software (Version 12, Stat-Ease, Inc.; Minneapolis, USA), employing a significance level of 0.05 in shake flask experiments.

## Results

### Effect of incubation time on carotenoid pigment production by *M. luteus*

**Fig. 1:**
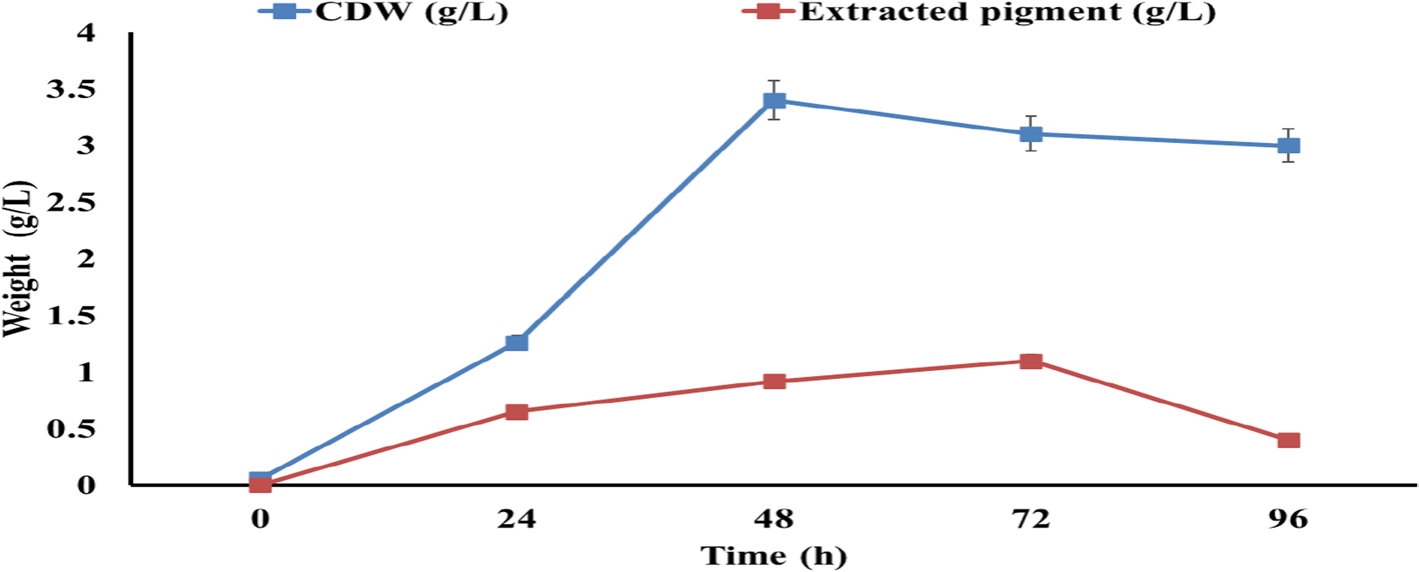
Carotenoid pigment production by *M. luteus* during 96 h of incubation at 30°C on whey media

Figure 1 shows the total carotenoid pigment production. The maximum total carotenoid pigment production reached 1.1 g/L after 72 h of incubation. This result aligns with the cell dry weight (CDW), which was 3.4 g/L. These data suggest that both cell growth and pigment production are most active up to 72 h, after which there is a noticeable decline in both parameters.

### Statistical screening of media components for carotenoid pigment production via the Box–Behnken design

**Fig. 2:**
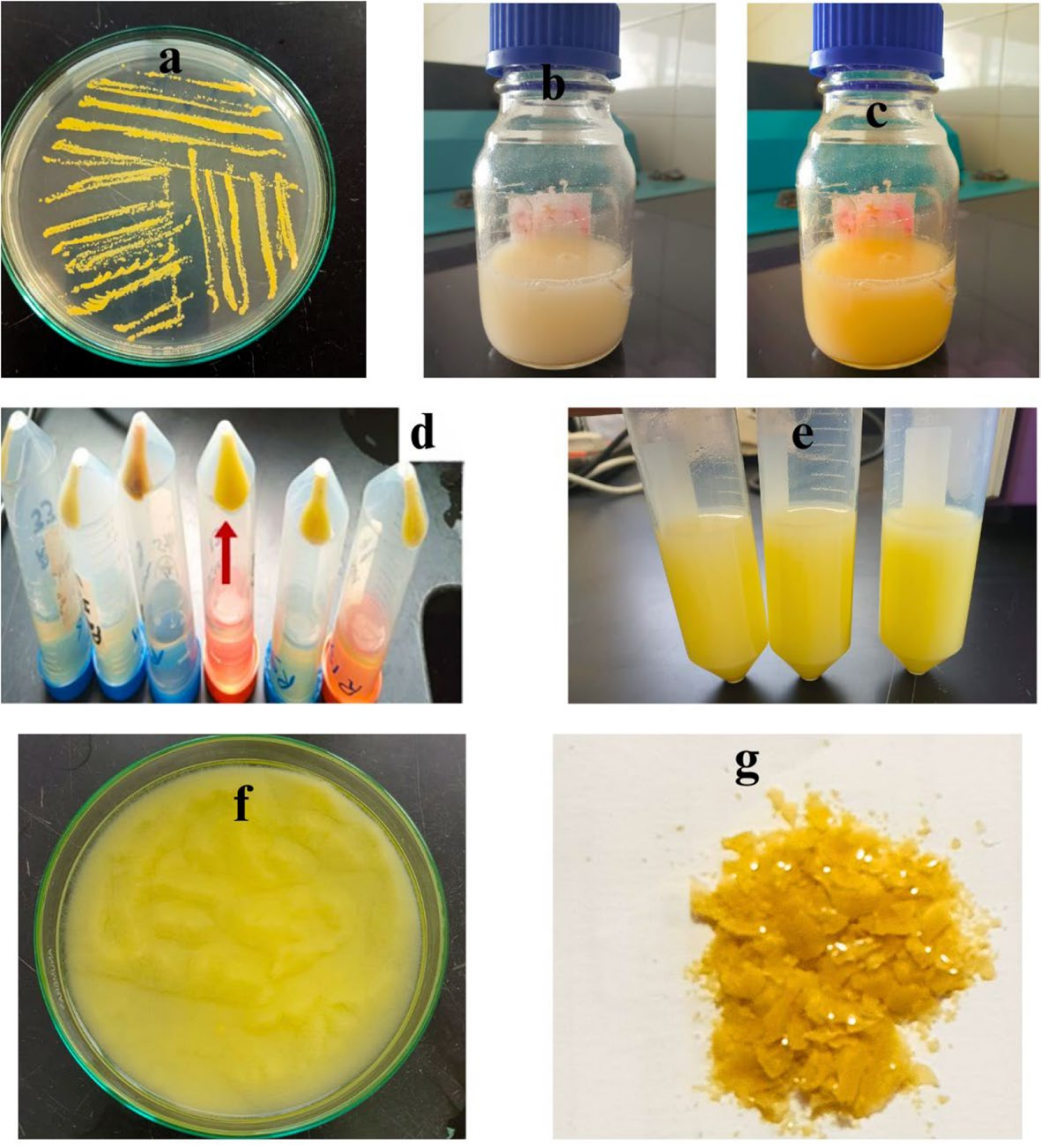
Box‒Behnken experimental runs for carotenoid production by *M. luteus* on whey media. **a** *Micrococcus luteus* culture; **b** Whey medium before and after carotenoid production; **c** different runs pointing to run 15 with red arrows; **d** extraction of carotenoids using ethanol; **e** carotenoid pigments during lyophilization; **f** and **g** carotenoid pigments after grinding

**Fig. 3:**
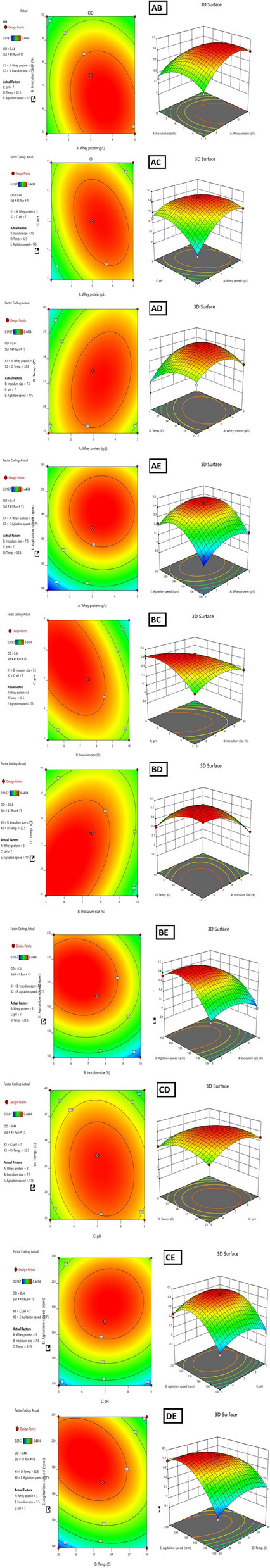
2D contour and 3D response surface plots of Box-Behnken design showing interactions between the different factors affecting carotenoid production using *M.luteus *AB: whey (g/L) and inoculum size (%), AC: whey (g/L) and pH, AD: whey (g/L) and temperature (°C), AE: whey (g/L) and agitation speed (rpm), BC: inoculum size (%) and pH, BD: inoculum size (%) and temperature (°C), BE: inoculum size (%) and agitation speed (rpm), CD: pH and temperature (°C), CE: pH and agitation speed (rpm), DE: temperature (°C) and agitation speed (rpm)

A BOX-Behnken design was employed in 46 test runs at both high and low levels for each variable to identify key factors influencing carotenoid pigment production. One-way ANOVA revealed the model's significance, with an F value of 47.02. Figure 2 outlines the various steps involved in the production and extraction of carotenoid pigments from microbial cultures. This includes microorganism growth (a), pigment formation in the culture medium (b, c), (solvent extraction of carotenoids (d), and lyophilization or drying of the extracted pigments (e, f), resulting in the final dried, powdered carotenoid pigment product (g). The main effects plot, shown in Fig. 3, is used alongside the ANOVA to determine the mean-level differences for all the variables. The study involved 46 experiments with different combinations of whey powder concentration (A), inoculum size (B), pH (C), temperature (D), and agitation speed (E), each tested at three levels, namely, − 1, 0, and + 1, with the actual and predicted responses presented. Table 1 shows that the maximum production (2.19 g/L) was achieved in runs 5, 15, 30, and 35 with the same levels of 3, 7.5, 7, 32.5, and 175 for the whey concentration (g/L), inoculum size (%), pH, temperature (°C), and agitation speed (rpm), respectively, under the conditions of 3% whey, 7.5% inoculum size, pH of 7, and agitation speed of 175 rpm at 32.5°C. The initial carotenoid production was 1.1 g/L, which increased to 2.19 g/L after optimization, representing a twofold increase. Figure 3 illustrates the surface response relationship between whey and other media components, showing how whey, inoculum size, pH, temperature, and agitation speed affect carotenoid pigment production, which led to a sharp increase in production by *M. luteus* cells to reach 2.19 g/L.

**Table 1:** BOX-Behnken design for carotenoid production and validation points for the significant model

Run order	Whey (A)	Inoculum size (B)	pH (C)	Temperature (D)	Agitation speed (E)	Carotenoid Experimental value (g/L)	Carotenoid Predicted value (g/L)
**1**	3	10	7	40	175	1.27	1.22
**2**	5	7.5	9	32.5	100	1.12	1.10
**3**	3	7.5	5	32.5	175	0.23	0.33
**4**	3	7.5	9	25	175	1.69	1.64
**5**	3	7.5	7	32.5	175	2.19	2.19
**6**	1	5	7	32.5	175	0.97	1.08
**7**	3	5	9	32.5	175	2.14	2.10
**8**	3	7.5	5	25	250	1.31	1.27
**9**	3	7.5	7	25	175	1.94	1.92
**10**	3	7.5	9	40	175	1.10	1.06
**11**	5	7.5	7	40	175	1.58	1.56
**12**	1	7.5	5	32.5	100	0.39	0.49
**13**	5	7.5	7	32.5	175	0.64	0.52
**14**	3	5	7	25	175	2.19	2.19
**15**	3	7.5	7	32.5	175	2.19	2.19
**16**	5	7.5	5	32.5	250	1.72	1.70
**17**	3	10	7	32.5	175	0.91	0.89
**18**	1	10	7	32.5	100	0.72	0.82
**19**	3	7.5	7	25	100	0.08	0.19
**20**	3	5	7	32.5	175	0.44	0.55
**21**	5	7.5	7	25	175	1.36	1.34
**22**	3	10	9	32.5	100	0.61	0.56
**23**	3	10	7	32.5	250	0.10	0.20
**24**	3	7.5	7	40	250	0.85	0.83
**25**	1	7.5	7	32.5	250	1.14	1.02
**26**	3	5	7	32.5	175	1.83	1.81
**27**	3	10	7	25	175	0.82	0.78
**28**	3	7.5	7	32.5	250	1.14	1.02
**29**	3	7.5	5	32.5	175	1.23	1.21
**30**	3	7.5	7	32.5	175	2.19	2.19
**31**	1	7.5	7	25	250	1.31	1.42
**32**	5	7.5	7	32.5	100	1.21	1.46
**33**	3	7.5	7	40	175	0.50	0.60
**34**	3	5	7	40	175	1.12	1.08
**35**	3	7.5	7	32.5	175	2.19	2.19
**36**	5	5	7	32.5	175	1.95	1.93
**37**	1	7.5	9	32.5	175	1.24	1.34
**38**	3	7.5	5	40	175	1.22	1.18
**39**	3	10	5	32.5	175	1.38	1.34
**40**	3	5	5	32.5	100	1.11	1.07
**41**	3	7.5	9	32.5	175	0.25	0.35
**42**	1	7.5	7	40	175	0.42	0.52
**43**	3	7.5	7	32.5	100	1.06	1.07
**44**	1	7.5	7	32.5	250	0.49	0.41
**45**	3	7.5	9	32.5	175	1.46	1.43
**46**	5	10	7	32.5	175	0.94	0.92
**Validation of the BOX model for medium components to optimize total carotenoid pigment production by ** ***M. luteus.***							
**Production parameters** **(and their levels)**^**a**^		Predicted value (g/L)		Observed value (g/L)	Biomass (g/L**)**	Productivity (gl^−1^h^−1^)	Productivity yield coefficient relative to biomass (g/g)
		**2.19**		2.19	3.4	0.045	0.644

Productivity (P) = amount of total carotenoid pigment produced (g/l)/fermentation time (h) = gl^−^1 h^−1^. Carotenoid pigment yield coefficient relative to biomass (Y_p/x_) (gg^−1^) = Amount of carotenoid pigment produced (g/l)/amount of biomass (gl^−1^)

^a^Production parameters: Whey powder concentration (3%), inoculum size (7.5%), pH (7), temperature (32.5 °C), agitation speed (175 rpm)

An F test and ANOVA were performed, with the model showing significance at an F value of 47.02. The model's coefficient of determination (*R* ^2^) was 0.9741, indicating that 97.41% of the total variation was explained by the model, indicating strong agreement between the experimental results and the predicted values. The final equation in terms of the actual factors is provided.

Y (total carotenoid pigment) = -5.7499 + 0.257905 * Whey + 0.229312 * Inoculum size + 0.572972 * pH + 0.0865806 * Temp. + 0.0174456 * agitation speed + -0.007565 * Whey * Inoculum size + -0.018125 * Whey * pH + 0.00376 * Whey * Temp. + -2.63333e-05 * Whey * Agitation speed + -0.018105 * Inoculum size * pH + 0.00417867 * Inoculum size * Temp. + -0.000152667 * Inoculum size * Agitation speed + -0.00167 * pH * Temp. + 7e-05 * pH * agitation speed + -0.000134089 * temperature. * Agitation speed + -0.0279479 * Whey^2 + -0.0142947 * Inoculum size^2 + -0.0238938 * pH^2 + -0.0015163 * Temp.^2 + -3.15467e-05 * Agitation speed^2. where Y represents the predicted response. Model validation confirmed that both the actual and predicted values were 2.19 g/L, yielding a productivity rate of 0.045 gL⁻^1^h⁻^1^ and a productivity yield of 0.644 g/g, as shown in Table 1.

### High-performance liquid chromatography analysis of carotenoids produced by *M. luteus*

The analysis of the carotenoids produced by *M. luteus* via HPLC (Table 2) revealed the following 12 microbial carotenoid pigments: neoxanthin (0.0026 g/mL), 13-cis lycopene (0.0026 g/mL), lycopene (0.0015 g/mL), 9-cis lycopene (0.0011 g/mL), zeaxanthin (0.0009 g/mL), β-carotene (0.0009 g/mL), lycoxanthin (0.0007 g/mL), cis-β-carotene (0007 g/mL), lycopene epoxide (0.0006 g/mL), tetra-dehydrocarotenoid (0.0006 g/mL), antheraxanthin diester (0.0006 g/mL), and lycophyll diester (0.0006 g/mL), with the indicated retention times and concentrations quantified on the basis of peak areas (Table 2). Neoxanthin and 13-cis lycopene were the pigments produced the most by *M. luteus*, followed by lycopene and 9-cis lycopene, whereas lycopene epoxide, tetra-dehydrocarotenoid, antheraxanthin diester, and lycophyll diester had the lowest pigments produced by *M. luteus*.

**Table 2:**
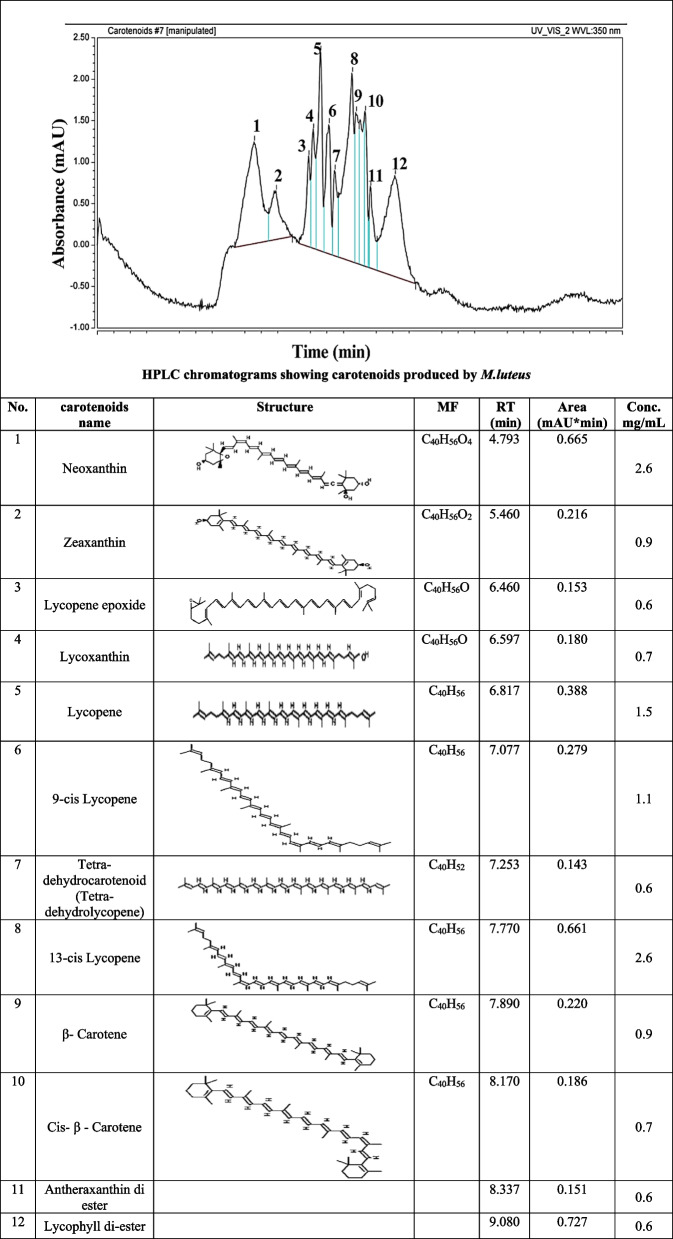
HPLC analysis of carotenoids produced by *M. luteus*

*RT* Retention time, *MF* Molecular formula

### Antimicrobial potential of carotenoids produced from *M. luteus*

Compared with that of the standard antibiotic tetracycline, the antimicrobial activity profile of carotenoids produced from *M. luteus* against pathogenic bacterial strains revealed notable trends, as shown in Table 3. Carotenoids have moderate activity against Gram-positive bacteria, such as *B. cereus*, with inhibition zone diameters between 17.0 and 29.0 mm; *S. aureus* (ranging from 0.0 to 14.0 mm); and *E. faecalis* (ranging from 28.0 to 37.0 mm), with inhibition zone diameter (IZD) values generally lower than those of tetracycline (35.0 to 45.0 mm) across varying concentrations. However, carotenoids demonstrate limited activity against Gram-negative bacteria, such as *S. typhi*, *E. coli*, and *S. sonnei*, with inhibition zone diameters of mostly 0.0 mm, particularly at lower concentrations, where tetracycline displays significant antimicrobial efficacy. Concentration dependency is evident for both carotenoids and tetracycline, with higher concentrations yielding larger IZD values. Statistical analysis confirmed significant differences in the IZD values among the concentrations. In conclusion, while carotenoids from *M. luteus* show promising antimicrobial activity against certain Gram-positive bacteria, their effectiveness lags behind that of tetracycline, especially against Gram-negative strains, emphasizing the importance of concentration optimization for maximizing their antimicrobial potential (Table 3).

**Table 3:** Antimicrobial activity of *M. luteus* carotenoids against pathogenic bacteria on NA at 30 °C for 24 h

	**Carotenoid (IZD) mm**					
	Gram-positive bacteria			Gram-negative bacteria		
**Conc. (µg/ml)**	*B. cereus*	*S. aureus*	*E. faecalis*	*S. typhi*	*E. coli*	*S. sonnei*
**1000**	29.0^e^ ± 0.15	14.0^ij^ ± 0.06	37.0^c^ ± 0.08	28.0^e^ ± 0.43	17.0^ h^ ± 0.18	20.0^ g^ ± 0.26
**500**	26.0^f^ ± 0.08	0.00	33.0^d^ ± 0.17	0.00	15.0^i^ ± 0.16	18.0^ h^ ± 0.25
**250**	21.0^ g^ ± 0.25	0.00	28.0^e^ ± 0.20	0.00	13.0^j^ ± 0.59	15.0^i^ ± 0.21
**125**	17.0^ h^ ± 0.30	0.00	0.00	0.00	0 .00	0.00
**75**	0.00	0.00	0.00	0.00	0.00	0.00
**0**	0.00	0.00	0.00	0.00	0.00	0.00
**Tetracycline (1000 µg/ml)**	35.0^d^ ± 0.38	45.0^a^ ± 0.27	40.0^b^ ± 0.26	45.0^a^ ± 0.30	38.0^c^ ± 0.41	40.0^b^ ± 0.34
**AI**	0.82	0.31	0.92	0.621	0.44	0.50

The same column is considered statistically nonsignificant in their variation

Tetracycline, used as the standard antibiotic, was tested sequentially against both Gram-positive (G + ve) and Gram-negative (G-ve) bacteria. The measurements are given in millimeters (mm), with the standard error (SE) indicated by the symbol ( ±). According to Tukey’s test at the 5% significance level, variables with the same letters denote statistically similar results

### Antioxidative activity of carotenoids produced from *M. luteus*

**Fig. 4:**
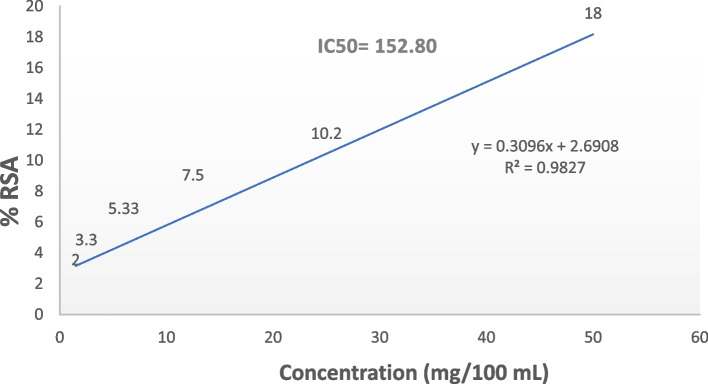
Free radical scavenging activity of carotenoids produced from *M. luteus* (expressed as % inhibition)

The antioxidant capacity of the carotenoid pigments from *M. luteus* was evaluated by measuring their ability to neutralize stable DPPH free radicals. The DPPH assay results, shown in Fig. 4, indicated that the antioxidant activities of the carotenoids at lower concentrations (1.5 and 2.5 mg/100 mL) were approximately 2.00% and 3.30%, respectively. At higher concentrations (6, 12.5, 25, and 50 mg/100 mL), the antiradical activity percentages were 5.33%, 7.50%, 10.20%, and 18.00%, respectively. The highest radical scavenging activity (RSA) of 18.00% was observed at a concentration of 50 mg/100 mL, with 10.20% at 25 mg/100 mL. An increase in the concentration of microbial carotenoid extracts corresponded to increased antioxidant activity, likely due to a greater amount of active pigments.

### Cytotoxicity of carotenoids produced by *M. luteus*

**Fig. 5:**
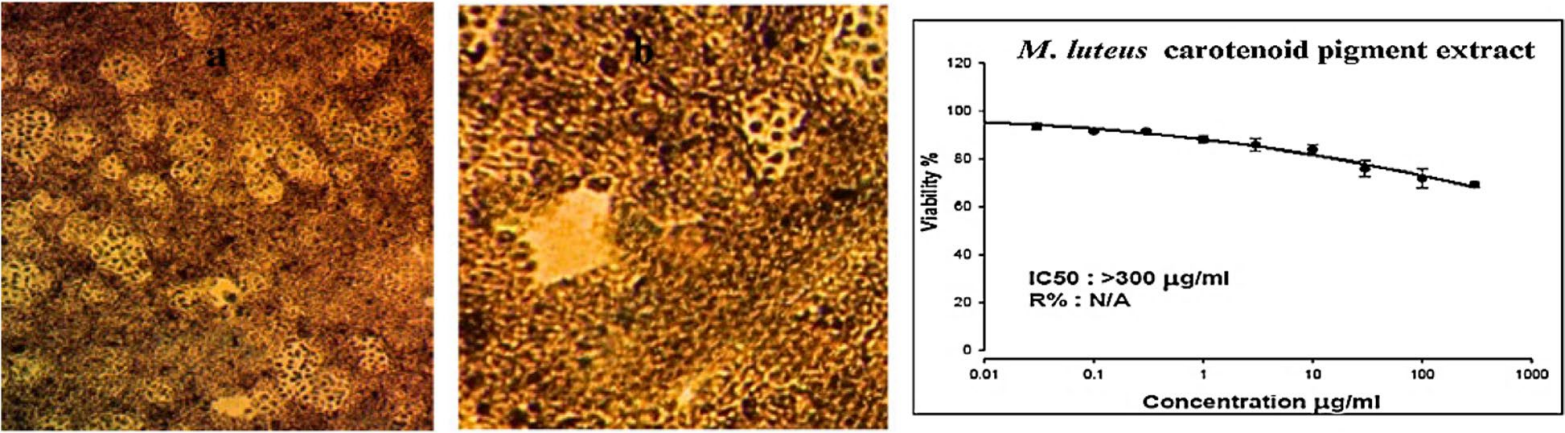
The cytotoxic effects of *M. luteus* carotenoid pigment**s** on normal mouse liver cells

Carotenoids produced by *M. luteus* were evaluated for their cytotoxicity against a normal mouse liver cell line, with tests conducted at concentrations ranging from 0.01 μg/mL to 300 μg/mL. Cell viability was assessed to determine the IC_50_ value. At the maximum concentration tested, 300 μg/mL, *M. luteus* carotenoid pigment treatment resulted in 68–71% cell viability. The IC_50_ value for the cytotoxicity of the *M. luteus* carotenoid pigment extract against normal mouse liver cells was greater than 300 μg/mL. Therefore, the carotenoid sample showed low toxicity against the normal liver cell line, with an IC_50_ exceeding the maximum tested concentration of 300 μg/mL. Over two-thirds of the liver cells survived treatment even at this high 300 μg/mL dose. In summary, the *M. luteus* carotenoid pigment extract demonstrated minimal cytotoxicity against normal mouse liver cells in vitro on the basis of the viability and IC_50_ data documented in Fig. 5.

### Histological studies of carotenoid pigments produced from *M. luteus*

**Fig. 6:**
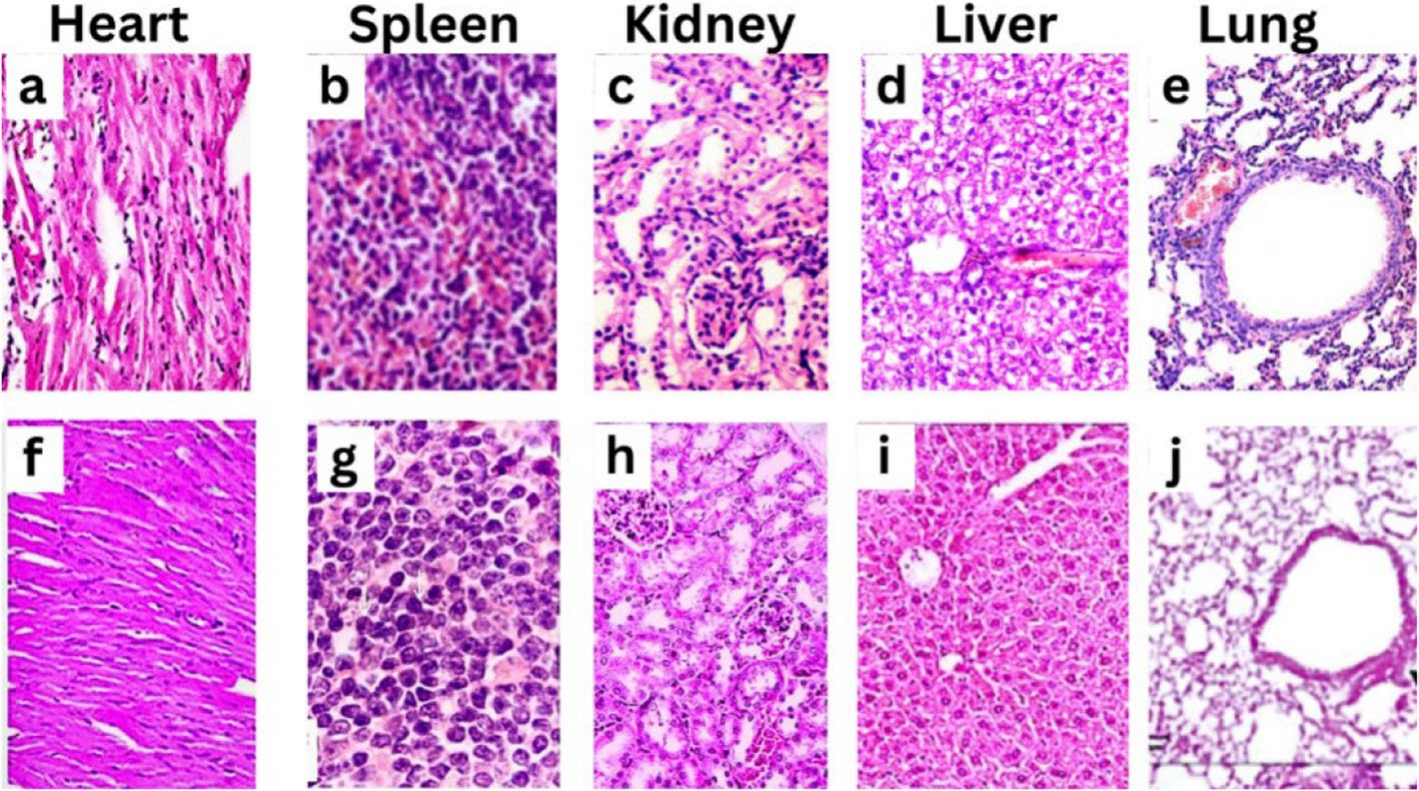
Histological results of heart, spleen, kidney, liver, and lung tissues after treatment with *M. luteus* carotenoids. Images **a**-**e** represent the control group (untreated) organs. Images **f**-**j** represent the corresponding organs treated with 100 μg/ml of the test substance. Microscopical examination was tested using 100X

Histological analysis, as shown in Fig. 6, revealed that the myocardial muscle tissue sections exhibited intramuscular edema and mild congestion of the intermuscular blood capillaries. The extent of myocardial muscle damage was classified as [(1) Mild—focal damage to myocytes or small, multifocal degeneration]. Lung tissue sections presented a normal histological structure, with airspaces separated by fine, delicate interalveolar septa; a normal vasculature with minimal perivascular connective tissue; folded columnar epithelial cells in the bronchioles; and a regular distribution of fibrous tissues. The alveoli were inflated with thin interalveolar septa, scoring 0. Splenic tissue sections displayed numerous round, elongated, or irregular lymphoid cell aggregates, known as white pulp, with well-organized periarteriolar lymphoid sheaths within the red pulp and clear, prominent margins, rated as (0). Kidney tissue sections revealed a normal histological appearance characterized by well-defined glomeruli with intact capillary tufts and Bowman's capsules. Both the proximal and distal convoluted tubules presented intact epithelial linings and a regular arrangement, scoring 0. Liver tissue sections showed ballooning degeneration of hepatocytes, disorganization of hepatic cords, Kupffer cell hyperplasia, and narrowing of hepatic sinusoids, graded as (II).

## Cytogenetic analysis of carotenoid pigments produced by *M. luteus*

Table 4 presents data from a study investigating the effects of different treatments (control, mitomycin C (MMC), and carotene) on various cytogenetic parameters in cells. The control group presented a normal mitotic index (80%), a low incidence of numerical chromosomal aberrations (0.6%) and no structural chromosomal aberrations. MMC, a known mutagen, significantly reduced the mitotic index (49.6%) and induced a high rate of numerical (17%) and structural aberrations (18.6%), including deletions, fragments, and centromeric attenuations. In contrast, the carotene treatment group presented a mitotic index (70%) lower than that of the control but higher than that of the MMC group and did not substantially increase the number (1.2%) or number of structural aberrations compared with that of the control group, suggesting a lack of genotoxic effects under the experimental conditions. The data were obtained by examining 10,000 cells for each treatment group.

**Table 4:** Statistical analysis of chromosomal aberrations in the bone marrow of male mice treated with MMC and carotene

	Treatments		
Parameter	Control	MMC	Carotene
Deletion	0.4 ± 0.24	11.2 ± 1.82	0.4 ± 0.24
Fragments	0.0 ± 0.0	1.6 ± 0.39	0.0 ± 0.0
Centromeric attenuations	0.0 ± 0.0	5.8 ± 1.35	0.0 ± 0.0
T. structural aberrations	0.4 ± 0.24	18.6 ± 1.69	0.0 ± 0.0
2n	49.4 ± 0.39	33 ± 1.22	48.8 ± 0.80
> 2n	0.4 ± 0.39	3.2 ± 0.86	0.8 ± 0.80
< 2n	0.2 ± 0.19	9.6 ± 2.29	0.4 ± 0.39
T. numerical aberrations	0.6 ± 0.39	17 ± 1.22	1.2 ± 0.80
No. examed cells	10000	10000	10000
Mitotic Index	80	49.6	70

The mean difference is significant at the .05 level

**Fig. 7:**
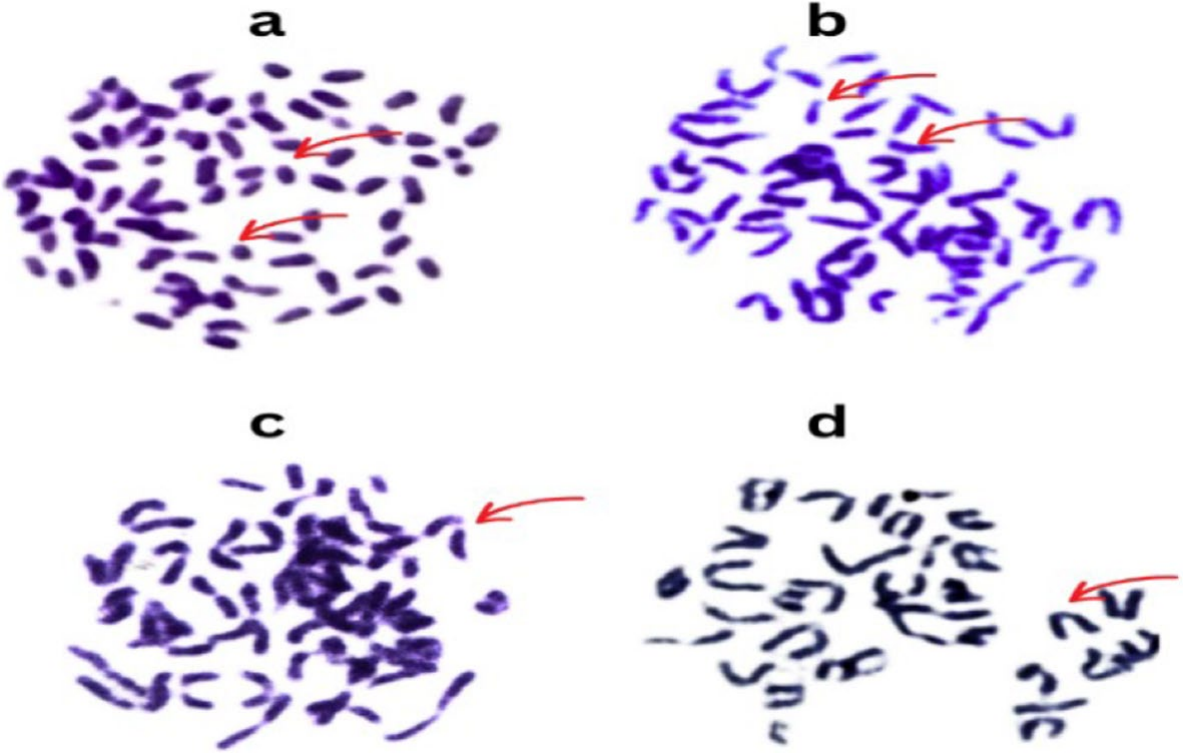
Metaphase spreads from the bone marrow of mice that were fed carotenoid pigments produced by *M. luteus*. **a** Normal metaphase spread, **b** fragments, **c** chromatid deletion, **d** centromeric attenuation

Cytogenetic analysis of carotenoid pigments produced from *M. luteus* (Fig. 7a-d) revealed centromeric attenuation in chromosomes with constricted or attenuated regions at the centromeres, indicating chromosomal instability or damage (Fig. 7a). From the results presented in (Fig. 7b), deletion was found. Illustrates chromosomes with portions missing or deleted, as indicated by the arrows pointing to the gaps or missing segments along the chromosome arms. While (Fig. 7c) shows fragments, the arrows in this image highlight smaller chromosome fragments that have been broken from the main chromosome structures. Figure 7d shows normal chromosomal spread without any apparent structural abnormalities, such as deletions, fragments, or centromeric attenuations.

## Discussion

Foods rich in carotene include fruits, vegetables, herbs, legumes, grains, and vegetable oils [27]. High levels of β-carotenes and lycopene are found in carrots, spinach, pumpkins, broccoli, sweet potatoes, tomatoes, apricots, and grapes [28]. Animal products, including salmon, trout, crabs, and poultry eggs, are the principal sources of xanthophylls in the human diet, including astaxanthin and canthaxanthin [29]. Aquaculture farming requires astaxanthin supplementation because the natural sources of salmonids are microalgae and zooplankton [16].

Carotenoids are produced industrially from natural sources such as plants, algae, and microbes or through chemical synthesis [30]. Synthetic carotenoids account for approximately 80–90% of the global market, with natural sources representing only a small fraction [31]. Natural carotenoids are found in microalgae such as *Haematococcus pluvialis* ([32] and *Dunaliella salina* [33]; fungi such as *Blakeslea trispora* [34]; yeasts such as *Phaffia rhodozyma* (*Xanthophyllomyces dendrorhous*) [35]; bacteria such as *Paracoccus carotinifaciens*; marigold flowers; and vegetables such as tomatoes and carrots. The most commonly used carotenoids on the global market are astaxanthin, canthaxanthin, zeaxanthin, lutein, β-carotene, and lycopene. Astaxanthin, β-carotene, and lutein together constitute 60% of the market value ([17].

The present study demonstrates the potential for optimizing carotenoid pigment production from *M. luteus* grown on whey media. The incubation time was found to significantly impact carotenoid yields, with the maximum production obtained at 72 h correlating with the highest biomass levels, indicating growth-associated biosynthesis. To further increase productivity, media components and physical parameters were screened systematically via a statistical Box–Behnken experimental design. Among the variables tested via response surface methodology (RSM) modelling, including the whey powder content, inoculum dose, pH, temperature, and agitation rate, the pH and inoculum level had the greatest influence. The carotenoid content increased sharply with increasing pH from 5 to 7 and stabilized at neutral values. However, alkaline pH had an inhibitory effect. Similarly, the inoculum dose was directly proportional to the carotenoid yield until it reached an optimum value of 7.5%, beyond which biomass accumulation resulted in negative feedback inhibition. Whey concentrations above 5% also led to a decline in pigment production, likely due to nutrient imbalance at higher whey loads.

By fitting the RSM data to a polynomial regression equation and analysing response surface plots, an optimized whey medium formulation and growth conditions could be defined. The Box–Behnken model enables the identification of interactions between variables and the prediction of responses under untested conditions within the experimental range. A composition of 3% whey and an inoculum dose of 7.5% at a neutral pH of 7, a moderate temperature of 32.5°C, and an agitation speed of 175 rpm was predicted and experimentally validated to increase the carotenoid titre to 2.19 g/L at 72 h. The statistical optimization strategy led to a 2-fold increase in carotenoid yields compared with whey production levels of 1.1 g/L with unoptimized parameters. Optimization increased the volumetric productivity to 0.045 g/L/h, with 0.644 g of pigment generated per gram of biomass. Overall, the model-guided sequential optimization approach enabled efficient improvement in microbial carotenoid biosynthesis.

The predominant carotenoids synthesized by *M. luteus* were elucidated via HPLC. Lycopene, zeaxanthin, and tetraterpenoid carotene derivatives were the primary compounds produced, resulting in bright yellowish-red hues. Carotenoids and apocarotenoids play vital roles as pigments in addition to their antioxidative and potential nutraceutical bioactivities, which merit investigation. Carotenoids such as β-carotene and canthaxanthin are frequently used as colorants in food products. Antioxidants are present in a range of foods, including fruit juices, beverages, pasta, chocolate, margarine, cheese, milk, sausages, and soft drinks. Lycoxanthin is a carotenol that is carotenol psi,psi-carotene substituted by a hydroxy group. It derives from a hydride of a lycopene. Epoxide xanthophylls like antheraxanthin and neoxanthin have pale yellow and dark yellow to orange colors. Antheraxanthin has antioxidant activity, it protects the cell from oxidative stress. As lycopene exhibits the greatest potential in oxygen quenching among all carotenoids, its isomers have been found to vary in their antioxidant properties, as well. 5-cis lycopene has been found to be most potent, followed by 9-cis. The weakest antioxidant properties have been reported for the all-trans isomer [36].

Carotenoids are isoprenoid compounds that serve as pigments in both photosynthetic and non-photosynthetic organisms. They are also crucial in biological systems because of their role as antioxidants. Joshi et al. [36] reported that microbial carotenoids are promising substitutes for synthetic carotenoids. Accordingly, the free radical quenching efficiency of the extracted *M. luteus* carotenoids was evaluated via a DPPH assay. An appreciable 18% RSA was attained at a 50 mg/100 mL dose, indicating moderate dose-responsive antioxidant activity, likely due to the conjugated polyene structures. Although lower than that of potent antioxidants such as BHT, this free radical neutralization capacity holds promise for alternative supplementation use. However, radical scavenging was limited to doses less than 3 mg/mL, restricting its efficacy for high-potency applications. Stereoisomerism plays a role in carotenoid antioxidant activity certain transforms have greater antioxidant activity than their cis counterparts, although exceptions such as cis-lycopene have greater bioavailability than trans-lycopene [36]. Our results revealed the production of carotenoid pigment from *M. luteus* grown on whey media consisting of C40 carotenoids, in the form cis. Therefore, carotenoids showed relatively low DPPH scavenging activity.

Several studies have demonstrated the limited efficacy of carotenoids against Gram-negative bacteria [37], they indicated that while carotenoids have moderate activity against Gram-positive bacteria and some fungi, their impact on Gram-negative bacteria is minimal. This was attributed to the protective outer membrane of Gram-negative bacteria, which inhibits the penetration of carotenoids due to the presence of lipopolysaccharide in the cell walls of Gram-negative bacteria that prevents entry of the pigment into the bacterial cells thus making it very resistant to pigments. The pigment extracts also exhibited selective antimicrobial effects against Gram-positive pathogens, including potent inhibition of *Enterococcus faecalis* and moderate suppression of Staphylococcal growth, with evident antilisterial effects [38].

Finally, to ascertain biosafety for prospective biotechnological and therapeutic utility, cytotoxic responses were investigated using a normal mouse hepatocyte line. Encouragingly, high cell survival even upon 300 μg/mL carotenoid treatment was documented, with over 2/3rd viability retained, confirming broad tolerability and low risk for hepatic injury. The negligible toxicity augments the feasibility of safe oral supplementation and topical or injectable formulation development aided by high IC_50_ values. However, the lack of examinations of cancerous and immune cell panels limits the overall safety of these methods[39]. Recent research has shed light on the potential anticancer properties of carotenoids, particularly those found in our extract. A study by [40] revealed that β-carotene, a major component of carotenoid extracts, exhibits anti-proliferative effects, promotes apoptosis, and induces cell cycle arrest in various triple-negative breast cancer (TNBC) cell lines, including MDA MB 231, MDA MB 235, and MCF7, when tested in vitro.

Furthermore, observed that combining β-carotene with doxorubicin [41] significantly lowered the inhibitory concentration required to combat breast cancer cells. Another investigation [42] found that β-carotene inhibited MCF7 cell line growth by enhancing antioxidant mechanisms. Beyond β-carotene, other key components of carotenoid extracts have shown promising results. Another study [43] conducted a series of in vitro and in vivo studies demonstrating that torulene and torularhodin possess anti-prostate cancer activities, primarily through the induction of apoptosis.

The histological examination of myocardial muscle tissue revealed signs of intramuscular edema along with mild congestion of intermuscular blood capillaries. This indicates a level of tissue damage, albeit categorized as mild with focal myocyte damage or small multifocal degeneration. These results align with earlier research that emphasized the histopathological alterations linked to myocardial injury [44]. In contrast, lung tissue sections presented a normal histological structure characterized by well-preserved alveolar architecture, intact vasculature, and normal epithelial cells lining the bronchioles. This suggests the absence of significant pathological changes in the lung tissue, which is crucial for respiratory function. Similar observations of normal lung histology have been reported in healthy individuals [45]. The splenic tissue sections showed typical features of white pulp and red pulp with well-defined periarteriolar lymphoid sheaths and clear demarcation between the two zones. These findings are consistent with previous descriptions of normal splenic histology. In the kidney tissue, histological examination revealed normal glomerular and tubular structures, indicating the absence of renal pathology. This aligns with the expected histological appearance of healthy kidneys. However, liver tissue sections exhibited ballooning degeneration of hepatocytes and disorganization of hepatic cords, indicating liver injury and pathological changes associated with hepatocellular damage. Comparable histological alterations have been noted in liver conditions such as nonalcoholic fatty liver disease (NAFLD) [46].

The cytogenetic analysis demonstrated significant differences among the treatment groups in terms of chromosomal aberrations. The control group presented minimal numerical and structural chromosomal aberrations, indicative of chromosomal stability under normal conditions. These findings are consistent with those of previous studies assessing baseline chromosomal integrity [47]. In contrast, treatment with mitomycin C (MMC), a known mutagen, led to a significant increase in both numerical and structural chromosomal aberrations, including deletions, fragments, and centromeric attenuations. These findings corroborate the genotoxic effects of MMC reported in numerous studies [48]. On the other hand, treatment with carotene did not induce substantial chromosomal aberrations compared with those in the control group, suggesting a lack of genotoxic effects. This finding aligns with the protective role of carotene, a potent antioxidant, against DNA damage and chromosomal instability [49].

## Conclusion

In conclusion, this study successfully increased carotenoid yields in whey-grown *M. luteus* nearly twofold when an economical substrate was used and optimized cultivation for high-density pigment production, with added commercial value. The statistical design tools enabled multivariate optimization to increase metabolite titres. HPLC revealed a rich diversity of health-beneficial carotenoid entities with appreciable free radical scavenging ability and selective antimicrobial effects against gram-positive food contaminants, although the effects were concentration dependent. Encouragingly, high tolerability to liver cells has expanded prospective utility. These histological and cytogenetic analyses provide valuable insights into tissue morphology and chromosomal integrity, highlighting the effects of different treatments on cellular structure and function. Overall, with further potentiation of functional bioactivities via purification or chemical modifications, the produced carotenoids have promising potential for alimentary, nutraceutical, and biopharmaceutical applications.

## Supplementary Information

Supplementary Material 1.

## Data Availability

The raw data and analysed data used during the current study are available from the corresponding author upon reasonable request. All microbial pathogens were provided by the Microbial Resources Centre Department, Faculty of Agriculture, Ain Shams University, Cairo, Egypt, and were deposited in the following strain providers: 1.Micrococcus luteus ATCC 9341 https://www.atcc.org/products/9341 2.Bacillus cereus ATCC 11778 https://www.atcc.org/products/11778 3.Staphylococcus aureus ATCC 6538 https://www.atcc.org/products/6538 4.E. faecalis ATCC 19433 https://www.atcc.org/products/19433 5.S. typhi DSM 17058 was obtained from the DSM collection https://www.dsmz.de/collection/catalogue/details/culture/DSM-17058 6.S. sonnei DSM 5570 was obtained from the DSM collection https://www.dsmz.de/collection/catalogue/details/culture/DSM-5570 7.E. coli ATCC 8739 was obtained from the ATCC collection https://www.atcc.org/products/8739.

## Abbreviations

MMC: Mitomycin
MIRCEN: Microbiological Resources Centre, Cairo, Egypt
DPPH: 1,1-Diphenyl-2-picrylhydrazyl
HSF: Human skin fibroblast
SRB: Sulforhodamine B
CDW: Cell Dry Weight
RT: Retention Time
MF: Molecular Formula
IZD: Inhibition Zone Diameter
IC50: Half-maximal inhibitory concentration
RSM: Response surface methodology
HPLC: High Performance Liquid Chromatography
RSA: Radical scavenging activity
BHT: Butylated hydroxytoluene
