# Supplementary material for: Optimization, characterization and biosafety of carotenoids produced from whey using Micrococcus luteus
Source: BMC Biotechnol. 2024 Oct 7;24:74. doi: 10.1186/s12896-024-00899-6 (PMC11459989; doi:10.1186/s12896-024-00899-6)
Supplement: Supplementary file 1 — Supplementary Material 1. [file 12896_2024_899_MOESM1_ESM.docx]

**Journal name:** [**BMC Biotechnology**](https://bmcbiotechnol.biomedcentral.com/)

**Manuscript title: Optimization, Characterization and Biosafety of Carotenoids Produced from Whey Using *Micrococcus luteus***

**The name of authors: Aml A. Hegazy^1†^, Samah H. Abu-Hussien^2*†^, Neima K. Elsenosy^3^, Salwa M. El-Sayed^4^, Mohamed Y. Abo El-Naga^1^**

**Authors’affliations and addresses:**

^1^Food Science Department, Faculty of Agriculture, Ain Shams University, Cairo, 11241, Egypt

^2^Agricultural Microbiology Department, Faculty of Agriculture, Ain Shams University, Cairo, 11241, Egypt

^3^Genetics Department, Faculty of Agriculture, Ain Shams University, Cairo, 11241, Egypt

^4^Biochemistry Department, Faculty of Agriculture, Ain Shams University, Cairo, 11241, Egypt

**^†^**Equal contribution authors

***Corresponding author**: Aml A. Hegazy

e-mail: [aml_hegazy@agr.asu.edu.eg](mailto:aml_hegazy@agr.asu.edu.eg)

phone number: +201067798412

**Table S1:** Levels of growth parameters tested in BOX-Behnken design

| **Variables** | **Symbols** | **Coded levels** | |
| --- | --- | --- | --- |
|  |  | -1 (Low) | +1 (High) |
| Whey | (A) | 7 | 3 |
| Inoculum size | (B) | 10 | 5 |
| pH | (C) | 5 | 9 |
| temperature | (D) | 25 | 40 |
| Agitation speed | (E) | 100 | 250 |

**Table (S2):** Box-Behnken design Matrix for growth parameters optimization experiment

| Run Order | Whey (A) | Inoculum size  (B) | pH  (C ) | Temperature (D) | Agitation speed (E) |
| --- | --- | --- | --- | --- | --- |
| 1 | 3 | 10 | 7 | 40 | 175 |
| 2 | 5 | 7.5 | 9 | 32.5 | 100 |
| 3 | 3 | 7.5 | 5 | 32.5 | 175 |
| 4 | 3 | 7.5 | 9 | 25 | 175 |
| 5 | 3 | 7.5 | 7 | 32.5 | 175 |
| 6 | 1 | 5 | 7 | 32.5 | 175 |
| 7 | 3 | 5 | 9 | 32.5 | 175 |
| 8 | 3 | 7.5 | 5 | 25 | 250 |
| 9 | 3 | 7.5 | 7 | 25 | 175 |
| 10 | 3 | 7.5 | 9 | 40 | 175 |
| 11 | 5 | 7.5 | 7 | 40 | 175 |
| 12 | 1 | 7.5 | 5 | 32.5 | 100 |
| 13 | 5 | 7.5 | 7 | 32.5 | 175 |
| 14 | 3 | 5 | 7 | 25 | 175 |
| 15 | 3 | 7.5 | 7 | 32.5 | 175 |
| 16 | 5 | 7.5 | 5 | 32.5 | 250 |
| 17 | 3 | 10 | 7 | 32.5 | 175 |
| 18 | 1 | 10 | 7 | 32.5 | 100 |
| 19 | 3 | 7.5 | 7 | 25 | 100 |
| 20 | 3 | 5 | 7 | 32.5 | 175 |
| 21 | 5 | 7.5 | 7 | 25 | 175 |
| 22 | 3 | 10 | 9 | 32.5 | 100 |
| 23 | 3 | 10 | 7 | 32.5 | 250 |
| 24 | 3 | 7.5 | 7 | 40 | 250 |
| 25 | 1 | 7.5 | 7 | 32.5 | 250 |
| 26 | 3 | 5 | 7 | 32.5 | 175 |
| 27 | 3 | 10 | 7 | 25 | 175 |
| 28 | 3 | 7.5 | 7 | 32.5 | 250 |
| 29 | 3 | 7.5 | 5 | 32.5 | 175 |
| 30 | 3 | 7.5 | 7 | 32.5 | 175 |
| 31 | 1 | 7.5 | 7 | 25 | 250 |
| 32 | 5 | 7.5 | 7 | 32.5 | 100 |
| 33 | 3 | 7.5 | 7 | 40 | 175 |
| 34 | 3 | 5 | 7 | 40 | 175 |
| 35 | 3 | 7.5 | 7 | 32.5 | 175 |
| 36 | 5 | 5 | 7 | 32.5 | 175 |
| 37 | 1 | 7.5 | 9 | 32.5 | 175 |
| 38 | 3 | 7.5 | 5 | 40 | 175 |
| 39 | 3 | 10 | 5 | 32.5 | 175 |
| 40 | 3 | 5 | 5 | 32.5 | 100 |
| 41 | 3 | 7.5 | 9 | 32.5 | 175 |
| 42 | 1 | 7.5 | 7 | 40 | 175 |
| 43 | 3 | 7.5 | 7 | 32.5 | 100 |
| 44 | 1 | 7.5 | 7 | 32.5 | 250 |
| 45 | 3 | 7.5 | 9 | 32.5 | 175 |
| 46 | 5 | 10 | 7 | 32.5 | 175 |
